# Predicting severe intraventricular hemorrhage or early death using machine learning algorithms in VLBWI of the Korean Neonatal Network Database

**DOI:** 10.1038/s41598-024-62033-y

**Published:** 2024-05-15

**Authors:** Hyun Ho Kim, Jin Kyu Kim, Seo Young Park

**Affiliations:** 1 Department of Pediatrics, Jeonbuk National University School of Medicine, Jeonju, South Korea; 2 Research Institute of Clinical Medicine of Jeonbuk National University-Biomedical Research Institute of Jeonbuk National University Hospital, Jeonju, South Korea; 3 Department of Statistics and Data Science, Korea National Open University, Seoul, South Korea

**Keywords:** Paediatric research, Computational biology and bioinformatics

## Abstract

Severe intraventricular hemorrhage (IVH) in premature infants can lead to serious neurological complications. This retrospective cohort study used the Korean Neonatal Network (KNN) dataset to develop prediction models for severe IVH or early death in very-low-birth-weight infants (VLBWIs) using machine-learning algorithms. The study included VLBWIs registered in the KNN database. The outcome was the diagnosis of IVH Grades 3–4 or death within one week of birth. Predictors were categorized into three groups based on their observed stage during the perinatal period. The dataset was divided into derivation and validation sets at an 8:2 ratio. Models were built using Logistic Regression with Ridge Regulation (LR), Random Forest, and eXtreme Gradient Boosting (XGB). Stage 1 models, based on predictors observed before birth, exhibited similar performance. Stage 2 models, based on predictors observed up to one hour after birth, showed improved performance in all models compared to Stage 1 models. Stage 3 models, based on predictors observed up to one week after birth, showed the best performance, particularly in the XGB model. Its integration into treatment and management protocols can potentially reduce the incidence of permanent brain injury caused by IVH during the early stages of birth.

## Introduction

In 2019, 41.5% of very-low-birth-weight infants (VLBWIs) registered in the Korean Neonatal Network (KNN) experienced intraventricular hemorrhage (IVH). Among these, 63.7% had severe IVH of Grade 3 or higher. IVH is associated with a poor prognosis, as evidenced by a modified Rankin Scale score of 4 to 6 upon discharge, and increases mortality risk by up to threefold^1^. IVH in preterm infants often occurs when multiple factors coincide or overlap, including changes in cerebral blood flow, abnormalities in autoregulation, and the vulnerability of the periventricular germinal matrix. This condition is associated with antenatal and postnatal clinical scenarios^2–5^. In other words, IVH in preterm infants most frequently occurs shortly after birth, and therapeutic options to dramatically improve the prognosis of established hemorrhage remain limited. Therefore, it is crucial to properly assess and manage the infant’s clinical conditions both before and after birth, with a special emphasis on IVH prevention.

The KNN was established in May 2013 with the support of the Korean Society of Neonatology and the Korea Centers for Disease Control and Prevention^6^. By October 2022, 77 of the approximately 100 neonatal intensive care units (NICUs) in South Korea had affiliated with the KNN. Every year, the KNN prospectively registers about 2,000–2,400 VLBWIs, representing 70%–80% of the total VLBWI population in the nation^7^. The data from the registered VLBWIs are being utilized as national big data for various research purposes.

The advancement of big data analytics has led to the use of artificial intelligence (AI), such as machine learning or deep learning, in various fields. AI has been extensively used in the medical field to develop prediction models for IVH based on imaging techniques such as cranial ultrasonography or brain magnetic resonance imaging. However, the majority of these studies have been conducted on adults^8,9^. In clinical studies on IVH based on the initial data from KNN, a national-level cohort, traditional statistical methods such as logistic regression have been used to identify risk factors^10^. While studies have used KNN data to analyze clinical factors in preterm infants using AI techniques such as machine learning and deep learning, no research has focused explicitly on factors associated with IVH until now^11,12^.

In this study, we aimed to use the KNN dataset to develop a predictive model for severe IVH or early death in VLBWIs using machine-learning algorithms.

## Methods

**Figure 1:**
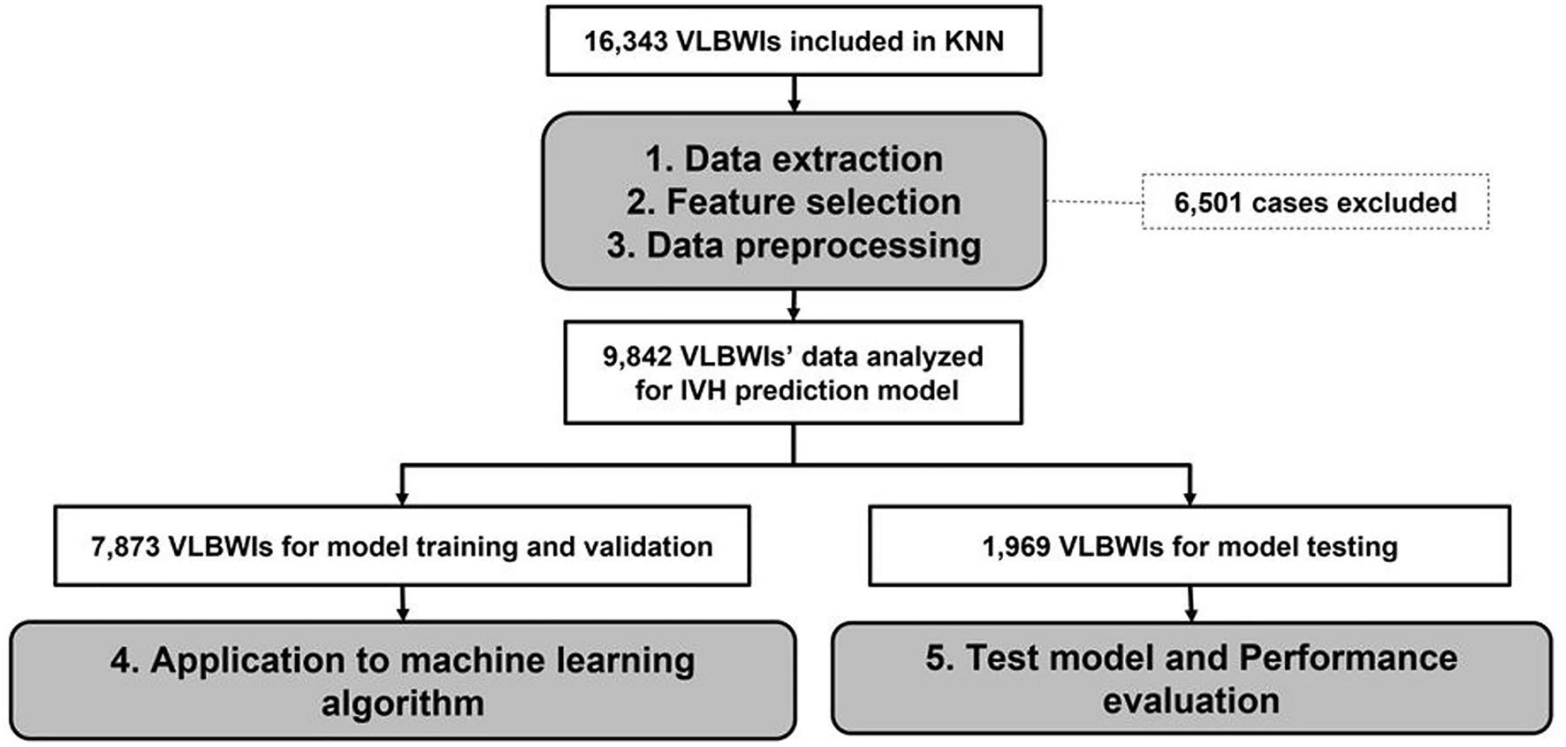
Flowchart of the study and data process. *VLBWIs* very low birth weight infants, *KNN* Korean Neonatal Network, *IVH* intraventricular hemorrhage.

A retrospective cohort study was conducted using the KNN registry database. The KNN registry had received approval from the Institutional Review Boards of each participating hospital, and prior consent was obtained from the parents of all infants when NICUs participating in KNN were registered. All methods were conducted in accordance with the approved Institutional Review Board protocol as well as related guidelines and regulations. Written informed consent was obtained from the parents or legal guardians of all participants involved in this study. After obtaining approval from the KNN’s Research Ethics Committee, data on VLBWIs were collected and analyzed^6^. The analysis included 16,343 VLBWIs with a birth weight of less than 1500 g, born in Korea and registered in the KNN between 2013 and 2020. However, 6501 infants with birth weights of less than 500 g, gestational ages less than 23 weeks, or missing data were excluded from the analysis (Fig. 1). All analytical processes, including data preprocessing, application of machine-learning algorithms, and performance evaluation, were conducted using Python Version 3.11.3.

Infants registered with the KNN have data collected during their NICU stay at the corrected ages of two and three years. The severity of IVH was determined based on the most severe findings observed in cranial ultrasonography conducted during the NICU stay. The stages of IVH were determined using the Papile classification system^13^. According to the study outcome, infants were categorized into two groups. Infants diagnosed with Stage 3 or 4 IVH or who died within the first week of life were labeled as “severe IVH/early death” or positive, while all other cases were labeled as negative.

Information acquired during the NICU admission period was used to formulate IVH-related input features available up to the first week after birth. In total, 22 variables from the KNN database were included. Prebirth data included the following: sex, the mother’s age, number of pregnancies, and in vitro fertilization status; maternal diabetes, hypertension, and clinical chorioamnionitis status; premature rupture of membrane duration; and delivery mode. Postbirth data were as follows: neonatal resuscitation stage, gestational age (GA), birth weight, Apgar scores at 1 and 5 min, and pH and base excess from arterial blood gas analysis performed within 1 h. Potential conditions during the first week after birth included pulmonary hemorrhage, respiratory distress syndrome, and hypotension requiring medication. The model’s performance was evaluated by categorizing the available information into three stages based on the timeline. Stage 1 models included feature values such as maternal and delivery information that could be obtained just before birth. Stage 2 models incorporated the input feature values from the first stage as well as delivery and neonatal information available within the first hour after birth. Stage 3 models incorporated the input features from the second stage as well as disease information diagnosable within the first week after birth (Table 1).

**Table 1:** List of input variables of the machine-learning models.

	Stage 1 model	Stage 2 model	Stage 3 model
Demographics	Sex and maternal age	Sex and maternal age	Sex and maternal age
Maternal information (before birth)	Multiple pregnancies, in vitro fertilization, maternal DM, maternal HTN, and chorioamnionitis	Multiple pregnancies, in vitro fertilization, maternal DM, maternal HTN, and chorioamnionitis	Multiple pregnancies, in vitro fertilization, maternal DM, maternal HTN, and chorioamnionitis
Delivery information (before birth)		Duration of PROM and mode of delivery	Duration of PROM and mode of delivery
Neonatal information (the first hour)		Neonatal resuscitation, GA, birth weight, 1-min and 5-min AS, and pH and base excess	Neonatal resuscitation, GA, birth weight, 1-min and 5-min AS, and pH and base excess
Disease information (the first week)			Pulmonary hemorrhage, respiratory distress syndrome, and hypotension requiring drugs

*GA* gestational age, *HTN* hypertension, *DM* diabetes mellitus, *AS* Apgar score, *PROM* premature rupture of membrane.

The dataset was divided into derivation and validation sets in 8:2 ratio to establish the machine-learning model. The dataset was randomly divided into variation and derivation with the “train_test_split” of splitting function, maintaining a consistent ratio of severe IVH/early death using a hyperparameter (Table 2). The derivation set was used for model training and hyperparameter selection, while the validation set was used to assess the model’s overall performance. To compare the baseline characteristics of neonates in the derivation and validation sets, the two-tailed *t*-test was used for continuous variables, the chi-squared test was used for categorical variables, and the significance of group differences was assessed using *p*-values and the standardized mean difference. Continuous predictors were normalized before entering the model by using the Min–Max scaling method. To adjust the range of numeric variable data points between 0 and 1 while maintaining their relative differences.

**Table 2:** Descriptive statistics for the derivation and validation sets.

Features	Total (*n* = 9842)	Derivation set (*n* = 7873)	Validation set (*n* = 1969)	*P*-value	SMD
Female, *n* (%)	4864 (49.4)	3901 (49.5)	963 (48.9)	0.63	0.01
Maternal age, mean (SD)	33.4 (4.2)	33.4 (4.2)	33.5 (4.2)	0.11	0.04
Multiple pregnancies, *n* (%)					
1	6216 (63.2)	4997 (63.5)	1219 (61.9)	0.17	0.05
2	3150 (32.0)	2512 (31.9)	638 (32.4)		
3	460 (4.7)	353 (4.5)	107 (5.4)		
≥ 4	16 (0.2)	11 (0.1)	5 (0.3)		
IVH grade, *n* (%)				0.21	0.07
No	5898 (59.9)	4733 (60.1)	1165 (59.2)		
1	2451 (24.9)	1950 (24.8)	501 (25.4)		
2	667 (6.8)	512 (6.5)	155 (7.9)		
3	293 (3.0)	239 (3.0)	54 (2.7)		
4	404 (4.1)	333 (4.2)	71 (3.6)		
Death within the first week, *n* (%)	129 (1.3)	106 (1.3)	23 (1.2)	1.00	0.01
Severe IVH or early death, *n* (%)	826 (8.4)	678 (8.6)	148 (7.5)	0.13	0.04
IVF, *n* (%)	2521 (25.6)	1996 (25.4)	525 (26.7)	0.25	0.03
Maternal DM, *n* (%)	1049 (10.7)	824 (10.5)	225 (11.4)	0.23	0.03
Maternal HTN, *n* (%)	2348 (23.9)	1864 (23.7)	484 (24.6)	0.42	0.02
Chorioamnionitis, *n* (%)	3166 (32.2)	2522 (32.0)	644 (32.7)	0.59	0.01
Duration of PROM, mean (SD)	2.3 (6.1)	2.3 (6.1)	2.3 (6.1)	0.76	0.01
C-section, *n* (%)	7925 (80.5)	6328 (80.4)	1597 (81.1)	0.48	0.02
Neonatal resuscitation, *n* (%)				0.70	0.04
O_2_	1147 (11.7)	930 (11.8)	217 (11.0)		
CPAP	1223 (12.4)	968 (12.3)	255 (13.0)		
PPV	2064 (21.0)	1643 (20.9)	421 (21.4)		
Intubation	5072 (51.5)	4055 (51.5)	1017 (51.7)		
Chest compression	135 (1.4)	110 (1.4)	25 (1.3)		
Epinephrine	201 (2.0)	167 (2.1)	34 (1.7)		
GA, mean (SD)	29.2 (2.9)	29.2 (2.9)	29.2 (3.0)	0.63	0.012
Birth weight, mean (SD)	1105.7 (268.2)	1105.2 (268.4)	1107.7 (267.6)	0.71	0.01
1-min Apgar score, mean (SD)	4.9 (2.0)	4.9 (2.0)	4.9 (2.0)	0.64	− 0.01
5-min Apgar score, mean (SD)	7.1 (1.8)	7.1 (1.8)	7.1 (1.7)	0.91	0.00
Birth weight, mean (SD)	1105.7 (268.2)	1105.2 (268.4)	1107.7 (267.6)	0.71	0.00
pH within 1 h, mean (SD)	7.3 (0.1)	7.3 (0.1)	7.3 (0.1)	0.09	0.04
Base access within 1 h, mean (SD)	− 5.2 (4.2)	− 5.2 (4.3)	− 5.0 (3.9)	< 0.05	0.07
Air leak syndrome, *n* (%)	419 (4.3)	335 (4.3)	84 (4.3)	1	0.00
Pulmonary hemorrhage, *n* (%)	460 (4.7)	367 (4.7)	93 (4.7)	0.96	0.00
Hypotension within the first week, *n* (%)	917 (9.3)	741 (9.4)	176 (8.9)	0.55	0.02
Respiratory distress syndrome, *n* (%)	7177 (72.9)	5755 (73.1)	1422 (72.2)	0.45	0.02

*SD* standard deviation, *hemorrhage IVH* intraventricular hemorrhage, *DM* diabetes mellitus, *HTN* hypertension, *PROM* premature rupture of membrane, *CPAP* continuous positive airway pressure, *PPV* positive pressure ventilation, *GA* gestational age, *SMD* standardized mean difference.

To address the imbalance in the target variable, we used the Synthetic Minority Over-sampling Technique (SMOTE) to over-sample the severe IVH/death group to a 1:1 ratio^14^. This approach generates additional samples from the minority class within the derivation data, thereby augmenting the dataset to facilitate enhanced balanced and effective learning process. The model’s training and tuning were conducted using the derivation data through stratified k-fold cross-validation^15^. The cross-validation for the training model was based on four iterations of the area under the receiver operating characteristic (AUROC). The models were constructed using classification machine-learning algorithms, including Logistic Regression with Ridge Regulation (LR), Random Forest (RF), and eXtreme Gradient Boosting (XGB). In this study, the grid search technique was used to optimize the hyperparameters^16^. The performance of the final models with selected hyperparameters was evaluated using the test dataset. To assess the performance of the binary classification models with the imbalance dataset, the AUROC, area under the precision-recall curve (AUPRC), weighted average F1-score, and accuracy were calculated based on the confusion matrix. Additionally, to identify the significance of clinical factors, the importance of input variables was examined in the machine-learning models.

## Results

A total of 9842 VLBWIs were included in the analysis. Among them, 826 infants (8.4%) experienced severe IVH/early death. The prevalence of severe IVH or early death was comparable between the derivation and validation sets, with 678 cases (8.6%) in the derivation set and 148 cases (7.5%) in the validation set (*p* = 0.13). In addition, other features did not differ significantly between the derivation and validation sets (Table 2).

The preprocessed predictor variables were categorized into three groups based on the timing of each observed variable. The predictors included features that could be obtained just before birth, such as demographic and maternal information. The severe IVH/early death group had a higher proportion of male and chorioamnionitis, as well as a lower proportion of maternal hypertension. Within an hour after birth, several characteristics showed significant differences depending on the target variable. Patients with severe IVH/early death had a lower frequency of cesarean section surgeries and required higher levels of neonatal resuscitation. They also had lower 1-min and 5-min Apgar scores and lower birth weight. Blood gas analysis within an hour after birth revealed lower pH and base excess values in this group. Additionally, in terms of diseases diagnosable within the first week after birth, there were significant differences in occurrence between infants with severe IVH/early death and those without. Air leak syndrome, pulmonary hemorrhage, hypotension, and respiratory distress syndrome were more frequently observed in the patient group with severe IVH/early death (Table 3).

**Table 3:** Features of the derivation set.

Features	Total (*n* = 7873)	No severe IVH and/or early death (*n* = 7195)	severe IVH and/or early death (*n* = 678)	*P*-value	SMD
Female, *n* (%)	3901 (49.5)	3621 (50.3)	280 (41.3)	< 0.05	0.18
Maternal age, mean (SD)	33.4 (4.2)	33.4 (4.2)	33.1 (4.4)	0.05	− 0.08
Multiple, *n* (%)				0.05	–
1	4997 (63.5)	4586 (63.7)	411 (60.6)		
2	2512 (31.9)	2268 (31.5)	244 (36.0)		
3	353 (4.5)	330 (4.6)	23 (3.4)		
≥ 4	11 (0.1)	11 (0.2)	0 (0.0)		
IVF, *n* (%)	1996 (25.4)	1815 (25.2)	181 (26.7)	0.43	0.03
Maternal DM, *n* (%)	824 (10.5)	766 (10.6)	58 (8.6)	0.10	0.07
Maternal HTN, *n* (%)	1864 (23.7)	1777 (24.7)	87 (12.8)	< 0.05	0.31
Chorioamnionitis, *n* (%)	2522 (32.0)	2229 (31.0)	293 (43.2)	< 0.05	0.26
Duration of PROM, mean (SD)	2.3 (6.1)	2.2 (6.0)	2.9 (7.1)	0.02	0.10
C-section, *n* (%)	6328 (80.4)	5835 (81.1)	493 (72.7)	< 0.05	0.20
Neonatal resuscitation, *n* (%)				< 0.05	0.97
O^2^	930 (11.8)	918 (12.8)	12 (1.8)		
CPAP	968 (12.3)	955 (13.3)	13 (1.9)		
PPV	1643 (20.9)	1598 (22.2)	45 (6.6)		
Intubation	4055 (51.5)	3532 (49.1)	523 (77.1)		
Chest compression	110 (1.4)	85 (1.2)	25 (3.7)		
Epinephrine	167 (2.1)	107 (1.5)	60 (8.8)		
GA, mean (SD)	29.2 (2.9)	29.5 (2.8)	26.3 (2.3)	< 0.05	− 1.24
1-min Apgar score, mean (SD)	4.9 (2.0)	5.1 (2.0)	3.3 (1.8)	< 0.05	− 0.92
5-min Apgar score, mean (SD)	7.1 (1.8)	7.2 (1.7)	5.6 (2.1)	< 0.05	− 0.86
Birth weight, mean (SD)	1105.2 (268.4)	1126.3 (259.9)	881.6 (254.8)	< 0.05	− 0.95
pH within one hour, mean (SD)	7.3 (0.1)	7.3 (0.1)	7.2 (0.2)	< 0.05	− 0.47
Base access within one hour, mean (SD)	− 5.2 (4.3)	− 5.0 (4.0)	− 7.8 (5.8)	< 0.05	− 0.57
Air leak syndrome, *n* (%)	335 (4.3)	264 (3.7)	71 (10.5)	< 0.05	0.27
Pulmonary hemorrhage, *n* (%)	367 (4.7)	225 (3.1)	142 (20.9)	< 0.05	0.57
Hypotension within the first week, *n* (%)	741 (9.4)	566 (7.9)	175 (25.8)	< 0.05	0.49
Respiratory distress syndrome, *n* (%)	5755 (73.1)	5108 (71.0)	647 (95.4)	< 0.05	0.69

*SD* standard deviation, *IVH* intraventricular hemorrhage, *DM* diabetes mellitus, *HTN* hypertension, *PROM* premature rupture of membrane, *CPAP* continuous positive airway pressure, *PPV* positive pressure ventilation, *GA* gestational age, *SMD* standardized mean difference.

**Figure 2:**
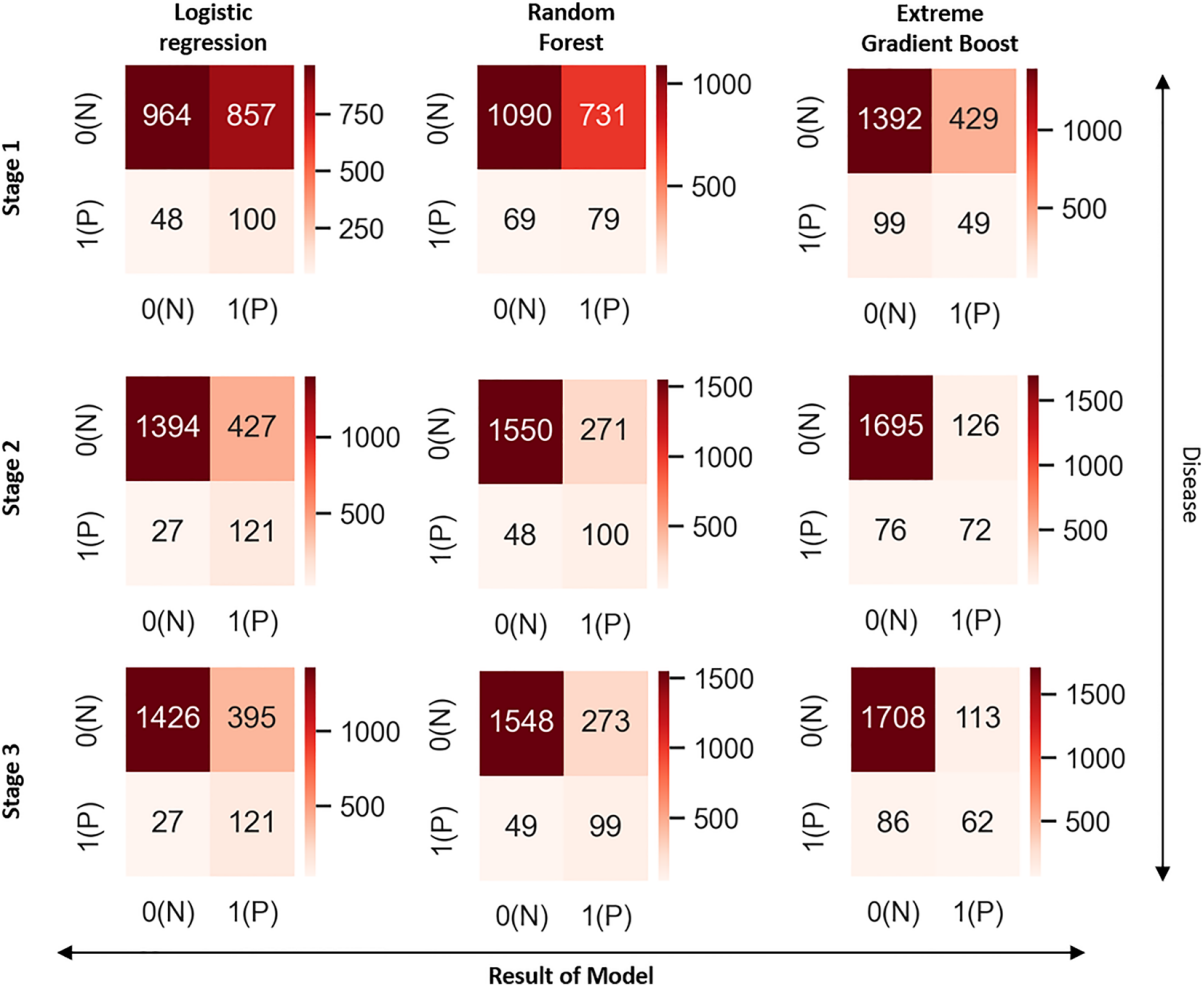
Confusion matrix of the validation set. *N* negative value, *P* positive value.

**Figure 3:**
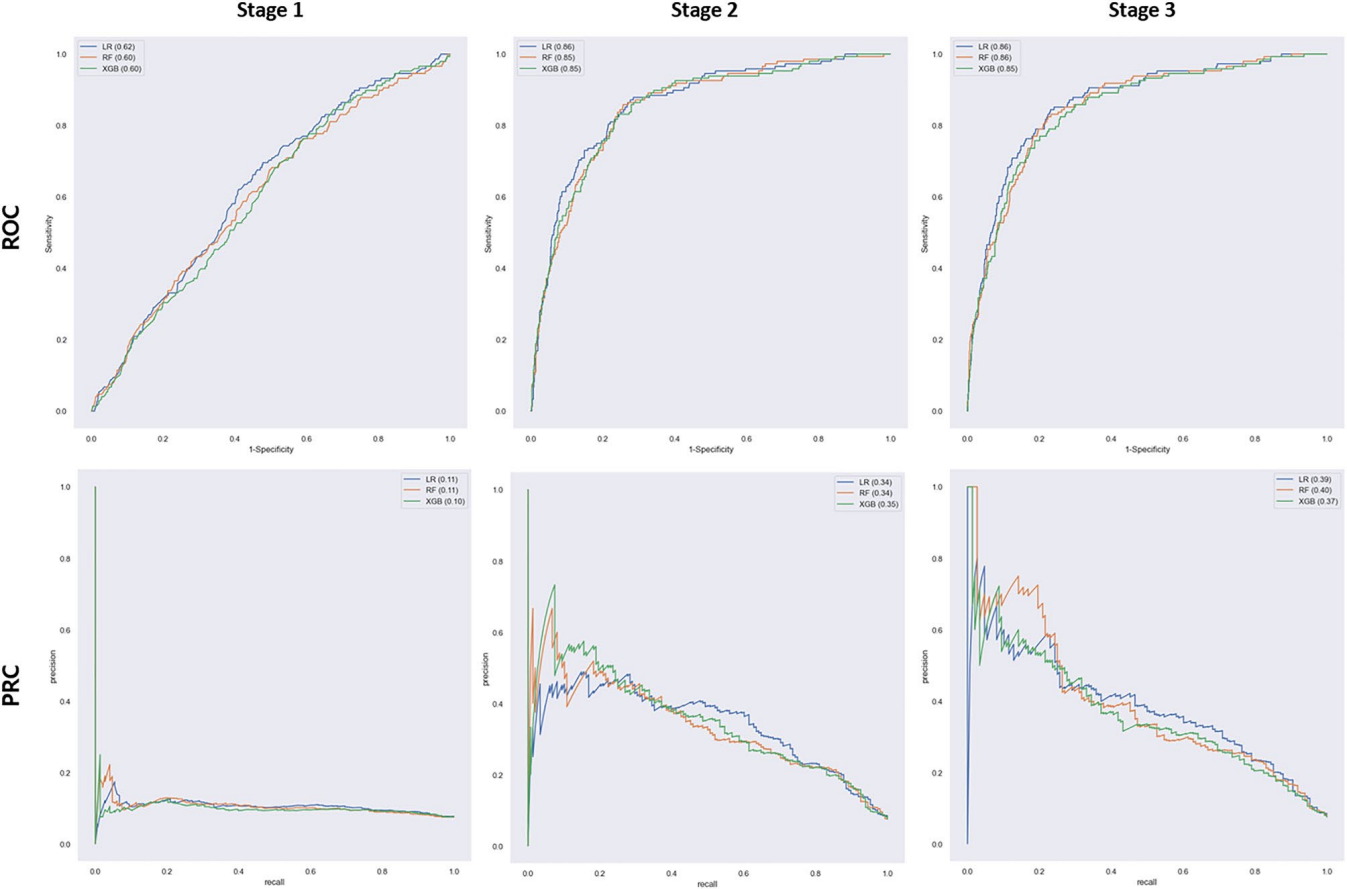
ROC and PRC curves of the IVH prediction models. *ROC* receiver operating characteristic, *PRC* precision-recall curve, *LR* logistic regression, *RF* random forest, *XGB* extreme gradient boosting.

We trained the model by employing SMOTE to oversample the severe IVH/death group, achieving a 1:1 ratio. Without oversampling, the predictive models tended to select cases without the disease, leading to a sensitivity close to zero and ultimately causing inadequate model training. In the Stage 1 model, the LR algorithm had the highest AUROC value of 0.62, but its weighted average F1 score of 0.64 was lower than that of other algorithms. The RF and XGB algorithms displayed AUROC values of 0.60, with corresponding weighted F1 scores of 0.69 and 0.79, respectively. Notably, the XGB algorithm achieved the highest accuracy of 0.73. The LR model exhibited a sensitivity of 67.6% in accurately predicting severe IVH/early death, whereas the XGB model demonstrated the highest specificity of 76.4% in predicting the normal groups. In the Stage 2 model, the algorithms emerged as good performers, with consistent AUROC values of 0.85 and 0.86. Notably, there was a significant improvement in the performance of all algorithms compared to the first stage, increasing their AUPRC from 0.11 to 0.34. Furthermore, the XGB algorithms achieved a consistent accuracy of 0.90. The sensitivity and specificity of all algorithms were improved, resulting in a significant improvement in the weighted average f1 score of LR from 0.64 to 0.82, RF from 0.69 to 0.87, and XGB from 0.79 to 0.90. In the Stage 3 model, the AUROC of all algorithms were ranged from 0.85 to 0.86. RF showed the best performance in AUPRC values with 0.40. Compared to Stage 2, Stage 3 showed a slight increase in the AUPRC of LR, but other than that, there was no difference in the performance of the Stage 2 model. In the final model, XGB showed the results of a predictive model with good overall performance (Table 4) (Figs. 2, 3).

**Table 4:** Performance results of the machine-learning model in the validation set.

Stage	Algorithm	AUROC	AUPRC	Weighted average F1 score	Accuracy
1	Logistic regression with ridge	0.62	0.11	0.64	0.54
	Random forest	0.60	0.11	0.69	0.59
	Extreme gradient boosting	0.60	0.10	0.79	0.73
2	Logistic regression with ridge	0.86	0.34	0.82	0.77
	Random forest	0.85	0.34	0.87	0.83
	Extreme gradient boosting	0.85	0.33	0.90	0.90
3	Logistic regression with ridge	0.86	0.39	0.83	0.79
	Random forest	0.85	0.40	0.87	0.84
	Extreme gradient boosting	0.85	0.37	0.90	0.90

*AUROC* area under receiver operating characteristic, *AUPRC* area under precision-recall curve.

**Figure 4:**
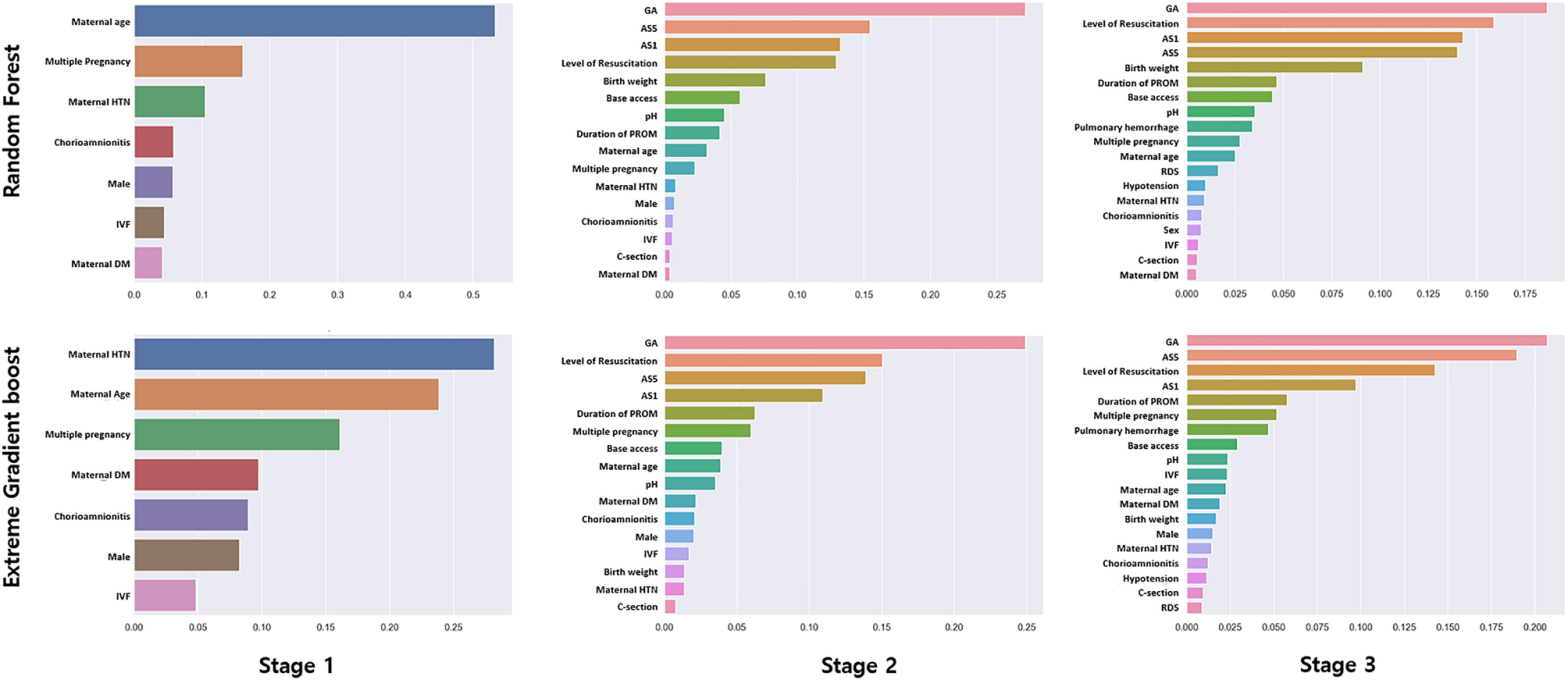
Feature importance of ensemble algorithms. *IVF* in vitro fertilization, *GA* gestational age, *AS* Apgar score, *PROM* premature rupture of membrane, *HTN* hypertension, *DM* diabetes mellitus, *RDS* respiratory distress syndrome.

We examined the feature importance of the ensemble model to assess its impact on severe IVH/early death. In the Stage 1 model, significant variables included maternal hypertension, maternal age, and multiple pregnancy. In Stage 2, variables such as GA, 1-min and 5-min Apgar scores, and level of neonatal resuscitation, which encompasses pre- and post-birth factors exhibited notable importance in the RF and XGB models. Additionally, the birth weight variable was significant in RF, whereas the duration of premature rupture of membrane was significant in XGB. Stage 3 showed similar findings to Stage 2, with newly added variables, including respiratory distress syndrome, pulmonic hemorrhage, and hypotension, showing less importance. This underscores the crucial role of perinatal factors, introduced in Stage 2, in predicting severe IVH/early death (Fig. 4).

## Discussion

In this study, we analyzed the Multi-institutional Neonatal Cohort Database, known as KNN. We developed a model to predict severe IVH or early death within the first week after birth using machine-learning algorithms. Our findings revealed that the XGB predictive model exhibited the highest performance in predicting the likelihood of severe IVH or death within the first week of infants’ lives. Compared to previous studies, our models showcased improved predictive capabilities with clinical information^17,18^. Additionally, from a clinical standpoint, our model’s applicability has expanded to encompass not only VLBWIs diagnosed with severe IVH but also infants who passed away within the first week after birth.

In a prospective study conducted in 2022, the incidence of severe stages 3–4 IVH was found to be 14.4%^18^. The difference in prevalence between this study (8.4%) and others is believed to be due to variations in cohort size and ethnicity. The data collected from the KNN, which covers the period from birth to discharge, is primarily managed by medical professionals and consists mainly of numerical data rather than time-series data. Therefore, we believe that research reflecting the interpretations and background knowledge of medical professionals based on these data can yield clinically applicable analytical results. Similar to predictive models for diseases with low incidence rates, it is essential to carefully construct a severe IVH/early death prediction model. In our approach, we used the SMOTE algorithm to oversample the IVH or early death groups and employed a stratified k-fold cross-validation technique during the model derivation process. To address the imbalance in the data, we examined AUPRC and weighted average f1 score alongside AUROC, focusing on interpreting the original data during the validation process.

Among various machine-learning algorithms, XGB ensemble algorithms using decision trees demonstrated the best performance. While conventional logistic regression data analysis relies on linearity, it has been established that various machine-learning algorithms, besides LR, can be effectively applied for data analysis, considering the increasing diversity of data information. Ensemble machine-learning algorithms, such as RF and XGB, which utilize decision trees, have improved their performance to a level comparable to the ridge-regularized LR algorithm in this study. Furthermore, by tailoring the application of machine-learning algorithms to the specifics of neonatal disease, it is plausible to establish an optimized predictive model for the disease.

In previous research on preterm infants, models have been constructed using logistic regression analysis. For instance, a single-institution prospective study published in 2022 from the Johns Hopkins Children’s Center’s NICU created a clinical model to predict severe IVH grade 3–4 at birth in 683 VLBWIs. This model demonstrated high performance, with an AUROC value of 0.83^18^. Similarly, a study conducted in 2013 based on the multi-national neonatal network, Neocosur Network, in South America developed a model using logistic regression analysis to predict severe IVH in 6,538 VLBWIs, achieving an AUROC value of 0.76^17^. Prior studies primarily analyzed medical data using linear algorithms such as linear and logistic regressions. However, in this research, the prediction performance was enhanced by applying the XGB ensemble machine-learning model, which has the ability to incorporate nonlinear relationships between the outcome and predictors. Research on neonatal IVH using AI is primarily being conducted in small, multi-institutional settings. For instance, in a retrospective study involving 265 preterm infants from two NICUs in Germany, early preterm infant brain hemorrhage was accurately predicted with approximately 90% accuracy using the RF machine-learning algorithm^19^. However, there are currently no studies that have proposed a model for predicting neonatal IVH based on national multi-institutional data.

In this study, the analysis of IVH/early death was segmented by time to develop models for each stage. It was observed that the performance notably improved in Stage 2 model with the introduction of newly incorporated variables obtained before and after birth. Variables such as GA and 1-min and 5-min Apgar scores, which are widely recognized as factors associated with severe IVH/early death, are routinely assessed in clinical practice for nearly all patients^20^. Additionally, level of neonatal resuscitation is a variable that aligns with the protocols outlined in clinical treatment guidelines immediately after birth^21^, thereby facilitating its integration into a predictive model while preserving the interventions performed by neonatologists. Notably, IVH is often attributed to a combination of multiple factors rather than a single clinical factor. Therefore, prediction models that encompass various clinical aspects of IVH may offer greater efficacy in clinical applications compared to those reliant solely on individual factors. Previous studies on prediction models using clinical factors have indicated that higher GA, 5-min Apgar score, hematocrit, and platelet count are associated with a reduced risk of IVH^18^. Because clinical factors related to pulmonary hemorrhage are also associated with low platelet count and hematocrit, these findings may have similar implications.

IVH is closely linked to mortality, particularly during the early stages of life. Given that brain ultrasound is typically conducted at first week after birth, instances of death occurring before confirming IVH may be inadvertently excluded from predictive models, potentially leading to predictions solely for surviving patients with IVH. Therefore, in this study, mortality was incorporated as a predictive variable to develop a model that could be effectively used in clinical practice. Machine-learning algorithms were applied to capture the temporal flow based on the data acquisition time, enabling a wide range of clinical applications. The Stage 1 model allows for the prediction of IVH or early death before delivery using data obtained prior to birth, enabling medical professionals to prepare for appropriate interventions in advance. The Stage 2 model is constructed based on data available from the time of delivery up to one hour later. By this time, emergency initial treatments following birth are generally completed, and the newborn enters a stabilization phase in the NICU. The predictive model for IVH at this stage can serve as an indicator for NICU management. The Stage 3 model is based on data available up to one week after birth. As more than 95% of preterm IVH cases occur within a week after birth, the Stage 3 model can be applied as an indicator for preventing severe IVH or early death.

Recently, Automated Machine Learning has been developed, and its utilization is expanding, enabling the simultaneous application of various machine-learning algorithms^22^. However, medical data requires a high level of expertise and insight, making the immediate application of Automated Machine Learning challenging. It is believed that further research is necessary to develop appropriate analytical techniques or machine-learning algorithms that are tailored to the specific characteristics of medical data. This study was designed to apply various algorithms for feasible clinical applications, highlighting their potential for diverse utility.

This study has several limitations that should be acknowledged. First, a significant portion of the total data was excluded from the study (39.8%) due to missing values. Future research could enhance the performance by using imputation algorithms to handle the missing values in the excluded data. Second, the analysis did not incorporate deep-learning techniques based on neural networks for feature extraction. Considering the characteristics of the numerical KNN data, generating new features based on the collected information and subsequently applying deep-learning techniques could potentially enhance the performance of the predictive model. Lastly, the KNN data analyzed in this study does not include information on race and mainly consists of Koreans. This may introduce a potential bias in the predictive model toward the Korean population. Therefore, future research should involve external validation with a more racially diverse patient population from various hospitals to ensure the generalizability and applicability of the predictive model.

A machine-learning algorithm has successfully developed the XGB model for predicting IVH and death within one week for VLBWIs. If the model is incorporated into treatment and management protocols, such as neonatal resuscitation, it has the potential to reduce the occurrence of permanent brain injury caused by IVH during the early stages of birth. Furthermore, it is believed that the medical records of the NICUs can be effectively utilized as a clinical decision support system.

## Data Availability

According to the KNN Publication Ethics Policy, the registered data is confidential and accessible only to researchers who have permission to access the research activities. The datasets generated and analyzed are not publicly available but are available from the corresponding author upon reasonable request.
